# Sparsened neuronal activity in an optogenetically activated olfactory glomerulus

**DOI:** 10.1038/s41598-018-33021-w

**Published:** 2018-10-08

**Authors:** Oliver Braubach, Tuce Tombaz, Tristan Geiller, Ryota Homma, Thomas Bozza, Lawrence B. Cohen, Yunsook Choi

**Affiliations:** 1 Center for Functional Connectomics, Brain Science Institute, Korea Institute of Science and Technology (KIST), Seoul, 136-791 Korea; 2 Department of Cellular and Molecular Physiology, Yale University School of Medicine, New Haven, CT 06510 USA; 3 NeuroImaging Cluster, Marine Biological Laboratory, Woods Hole, MA 02543 USA; 4 Department of Neurosurgery, Cedars Sinai Medical Institute, Los Angeles, CA 90048 USA; 5 Department of Neurobiology, Northwestern University, Evanston, IL 60208 USA; 6 Visiting Scientist Program, HHMI Janelia Farm Research Campus, Ashburn, VA 20147 USA

## Abstract

Glomeruli are the functional units of olfactory information processing but little remains known about their individual unit function. This is due to their widespread activation by odor stimuli. We expressed channelrhodopsin-2 in a single olfactory sensory neuron type, and used laser stimulation and simultaneous *in vivo* calcium imaging to study the responses of a single glomerulus to optogenetic stimulation. Calcium signals in the neuropil of this glomerulus were representative of the sensory input and nearly identical if evoked by intensity-matched odor and laser stimuli. However, significantly fewer glomerular layer interneurons and olfactory bulb output neurons (mitral cells) responded to optogenetic versus odor stimuli, resulting in a small and spatially compact optogenetic glomerular unit response. Temporal features of laser stimuli were represented with high fidelity in the neuropil of the glomerulus and the mitral cells, but not in interneurons. Increases in laser stimulus intensity were encoded by larger signal amplitudes in all compartments of the glomerulus, and by the recruitment of additional interneurons and mitral cells. No spatial expansion of the glomerular unit response was observed in response to stronger input stimuli. Our data are among the first descriptions of input-output transformations in a selectively activated olfactory glomerulus.

## Introduction

Mouse olfactory bulbs contain approximately 2000 glomeruli that are each innervated by sensory neurons expressing a single functional odorant receptor type^1,2^. The molecular receptive range of odorant receptors is extensive, and sensory neurons may respond to many odors^3–6^. Glomeruli and downstream neurons therefore respond with complex and overlapping activation patterns to simple odor stimuli^7–10^. The outputs of a single glomerulus are carried by 20–25 mitral/tufted cells^11,12^, and each mitral cell projects axons to vast areas of the brain^13^. Trying to decipher the input-output logic of the olfactory bulb is thus very complicated, especially when a single odor activates many glomeruli. Yet, we do know that a single glomerulus can relay sufficient neural information to elicit a learned behavioral response^14^. We thus sought to establish an experimental model of single glomerular activation, and to clarify some aspects of the input-output logic of the mouse olfactory bulb.

Glomeruli filter and control the transmission of incoming odor stimuli to downstream brain regions. The transfer of information between sensory neurons and postsynaptic mitral/tufted cells is modulated by hundreds of local interneurons, including GABAergic periglomerular cells^15^, short axon cells^16,17^, and external tufted cells^18^. GABAergic periglomerular cells control the excitability of individual glomeruli via tonic and feedback inhibition of olfactory sensory neuron axon terminals^19–22^, and exert feedforward inhibition on mitral cell dendrites^23^. Olfactory representations may also be shaped by interglomerular interactions. Center surround^16,24,25^ and distance-independent^15,26,27^ interactions have been observed *in vitro*. *In vivo* data suggest that a global center-surround inhibitory process^28^, or specific inhibitory interactions among similarly tuned adjacent glomeruli^29^ are the primary modes of glomerular interactions. However, the extent of these lateral glomerular interactions remain unclear, and this may be due to the fact that there are few or no glomeruli that respond *selectively* to an odor stimulus. Rather, odor-evoked glomerular activity is widespread and presumably drives parallel and/or competing lateral interactions among glomeruli. Thus, there is a need to establish a physiological model for single glomerular activation.

We established an *all-optical* experimental strategy to activate and record activity from a single glomerulus *in vivo*. We investigated (1) how glomeruli respond to odor vs. optogenetic activation, (2) how optogenetic stimuli are represented in the glomerular neuropil, interneurons and mitral cells, and (3) how stimulus intensity is encoded in the optogenetically-activated glomerulus. We find (1) a significant reduction in the amplitude(s) and population size(s) of cells responding to laser vs. odor stimuli; (2) discrete temporal stimulus representations across different compartments of the glomerulus, and (3) describe a glomerular unit as a very compact population of interneurons and an output compartment that is made up of very few mitral cells.

## Results

**Figure 1:**
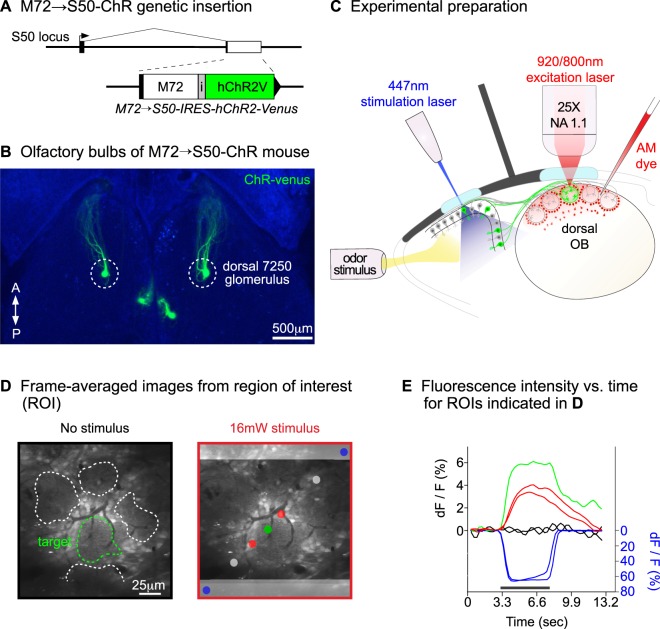
Transgenic mouse and overview of the experimental method. (**A**) Design of the gene targeted allele. The S50 coding sequence (white box) was replaced with that of M72, followed by an internal ribosome entry site (i) and the gene for channelrhodopsin-2 fused to Venus YFP. (**B**) Olfactory bulbs from a homozygous M72 → S50-ChR mouse. Channelrhodopsin-2 expressing glomeruli (7250 target glomeruli) were located on medial and dorsal olfactory bulb surfaces. Experiments were conducted with dorsal target glomeruli. (**C**) Experimental arrangement. The olfactory bulbs of head-fixed mice were imaged with a two-photon microscope. Neuronal activity was measured with a bolus-loaded AM calcium dye (red). Odor stimuli were delivered via an olfactometer nosepiece and laser stimuli were delivered through a window installed over the olfactory epithelia. Channelrhodopsin-2 expression is indicated in green. A black cardboard screen was installed between the olfactory epithelium and olfactory bulb to block stimulation laser light from reaching the microscope lens. (**D**) Stimulation sequence used to eliminate optical interference from the laser stimulus. The left panels shows a Fura-loaded imaging region (no stimulation). The neuropil of each glomerulus is outlined. The right panel shows the average image during 447 nm laser stimulation (16 mW), delivered as 18 × 60 msec pulses separated by 305 ms intervals. Optical interference is visible on the top and bottom of the frame as light grey horizontal bars. (**E**) Optical signals from regions of interest from the right panel in (**D**). Blue traces show the optical interference from the stimulation laser; this interference did not penetrate the central region of the image where a glomerulus (green), glomerular layer interneurons (red) and non-responding regions (black-grey) were located. The scale bar in the left panel of (**D**) applies to both images.

We created transgenic mice that express channelrhodopsin-2 in M72 olfactory neurons (Fig. 1A and Methods). The M72 sensory neurons innervate two pairs of glomeruli, which we call the 7250 target glomeruli (Fig. 1B). Using the method illustrated schematically in Fig. 1C, we studied the neuronal activity associated with a single dorsal target glomerulus (see Methods). We measured three types of calcium signals. (1) Calcium signals in the glomerular neuropil were treated as ‘input’ signals because they originate mainly from axonal inputs to the glomeruli^30^. (2) Interneuron signals were treated as ‘modulation’ signals and were obtained from cell bodies located in the glomerular layer. (3) ‘Output’ signals were recorded from mitral cells located 150–200 μm below the olfactory bulb surface.

### Optogenetic vs. odor evoked activation of the 7250 target glomerulus

#### Comparing neuronal activity in response to odor vs. laser stimulation

Ethyl tiglate was administered at 0.02% to 10% of saturated vapor and was the only odor used to produce the results in this paper. Odor stimuli, when used, thus refers to ethyl tiglate. Laser stimuli were delivered to the olfactory epithelia at 0.048 mW to 16 mW average intensities. Preliminary experiments showed that 8.8 mW stimuli elicited the strongest calcium signals in the upstream glomeruli (Supplemental Fig. 1A). Higher stimulation intensities did not increase the amplitude of the glomerular calcium signal but instead triggered non-specific neuronal activity that was measurable in wild type mice (Supplemental Fig. 1B). We therefore capped average laser stimulation intensities at 16 mW. The smallest detectable glomerular signals were evoked by laser stimuli ranging from 0.048 to 0.16 mW average intensity (Supplemental Fig. 1A). Below, we refer to 10% ethyl tiglate vapor and 8.8 mW laser stimuli as strong stimuli; weak stimuli are 0.02% ethyl tiglate vapor and 0.16 mW laser stimuli.

#### Consistent odor and laser-evoked signals in the neuropil of the 7250 target glomerulus

**Figure 2:**
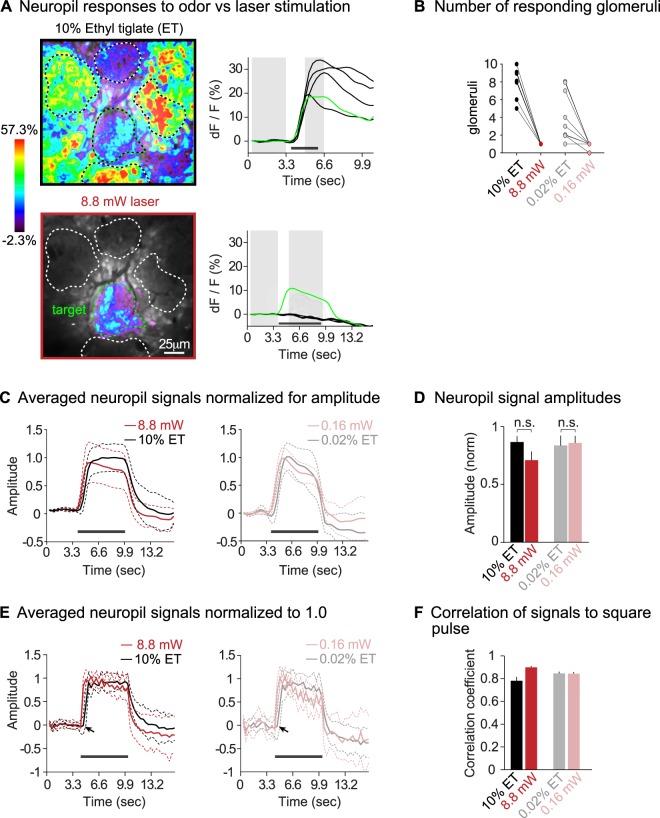
Neuropil signals in the 7250 target glomerulus during ethyl tiglate and laser stimulation (stimulus intensities as indicated). (**A**) Frame subtractions (left) and fluorescence intensity vs. time traces (right) showing odor vs. laser-evoked neuronal activity. Glomeruli are indicated with dashed lines; the target glomerulus is green. Grey bars in the right panels of (**A**) indicate the frames that were used to create the frame subtractions. The scale bar on the left applies to top and bottom panels. (**B**) The number of glomeruli that respond to odor vs. laser stimuli; responses were recorded in imaging frames sized 205–335 μm^2^. (**C**) Average calcium signals from multiple target glomeruli. The signals were normalized by dividing traces from ethyl tiglate and laser stimulation trials by the mean fluorescence intensity during the evoked response at 4.6–9.2 sec. Dashed lines indicate standard deviations. (**D**) Histogram comparing signal amplitudes in the neuropil; data were averaged and normalized as in (**C**). (**E**) Neuropil calcium signals that were normalized by setting the maximum of each trace to 1.0 to reveal the signal shapes (no temporal smoothing). The laser-evoked response has a slightly shorter latency than the odor-evoked response onset (arrow). (**F**) Histogram showing correlations of odor and laser-evoked calcium signals to the shape of the square command pulse for each stimulus (determined by measuring the Euclidian distance between the stimulus command pulse and the calcium signals). The black line in (**A**,**C**,**E)** indicates the duration of the stimulus command pulse. The abbreviation ET denotes the odor ethyl tiglate.

Exposing mice to 10% ethyl tiglate vapor elicited robust calcium signals in the 7250 target glomerulus and in surrounding interneurons, as well as other glomeruli in the imaging area. This is shown in the frame subtraction in the top left panel of Fig. 2A. This frame subtraction was created by subtracting the average of 10 frames (3.0 sec) before the stimulus presentation from 5 frames (1.5 sec) during the stimulus presentation; these frames are indicated with grey bars in the calcium signal traces in the right panel of Fig. 2A. The fluorescence intensity vs. time traces in the top panel of Fig. 2A show that every glomerulus in the imaging frame responded to 10% ethyl tiglate vapor with a strong calcium signal. In contrast, laser stimulation of channelrhodopsin-2 expressing olfactory sensory neurons produced calcium signals only in the 7250 target glomerulus. This is shown in the frame subtraction and accompanying fluorescence intensity traces in the bottom panels of Fig. 2A. In 16 mice in which we monitored 5–10 glomeruli per imaging frame, we recorded a laser-evoked target glomerulus response in all but one specimen. We never observed a laser-evoked signal in any other glomerulus (Fig. 2B). Our laser stimulation method is thus selective and produces activation of just the 7250 target glomerulus.

Averaged calcium signals from the 7250 target glomerulus during ethyl tiglate vs. laser stimulation are shown in Fig. 2C. Each signal is shown as a fraction of the largest recorded signal in odor vs. laser stimulation measurements. Hence, if an odor-evoked signal was larger than a laser-evoked response, then the corresponding laser signal would be a fraction of the odor response (and vice versa). We observed no significant differences in the neuropil signal amplitudes evoked by ethyl tiglate vs. laser stimuli in strong (Fig. 2D; t(15) = 1.359, p = 0.19, paired t-test) and weak stimulus conditions (Fig. 2D; t(8) = 0.166, p = 0.87, paired t-test). Furthermore, laser-evoked signals in the target glomerulus rarely exceeded odor-evoked signals in neighboring glomeruli: the average laser-evoked signal in the 7250 target glomerulus was 46 ± 4% of the largest odor-evoked signal in adjacent glomeruli.

In addition to having the same amplitudes, laser and ethyl tiglate-evoked neuropil signals also had similar temporal profiles. Figure 2E shows average neuropil calcium signals without temporal smoothing to reveal the signal time course. To normalize amplitude differences, we set the peak amplitude of each trace equal to 1.0; the signal peaks weren’t always located in the same position(s) along the signal time course so that the averaged neuropil signal generally falls below 1.0 maximum amplitude. Overall, the calcium signals for ethyl tiglate and laser stimulation conditions were very similar (Fig. 2E). We correlated the time course of these signals to square pulses resembling the command signals for odor or laser stimuli, and observed similar, high correlation coefficients in each case (Fig. 2F). This implies that both, ethyl tiglate vapor and laser-evoked signals have approximately ‘square pulse shapes’ that are representative of the input stimuli. Nevertheless, laser-evoked responses had slightly faster onsets than odor-evoked responses: the average signal onset for laser responses was 629 ms, whereas odor responses where at 1067 ms (t(15) = 3, p = 0.009, paired t-test for strong stimulus conditions; arrows in Fig. 2E). We attribute this difference to the slower arrival of the ethyl tiglate vapor at the sensory epithelium. We conclude that laser stimulation of channelrhodopsin-2 expressing sensory neurons produced neuropil signals that were similar to signals evoked by ethyl tiglate vapor. Laser stimulation of the olfactory epithelium thus allowed us to mimic the sensory input that would normally be achieved by an odor stimulus.

#### Reduced cellular activation in the light-activated target glomerulus

**Figure 3:**
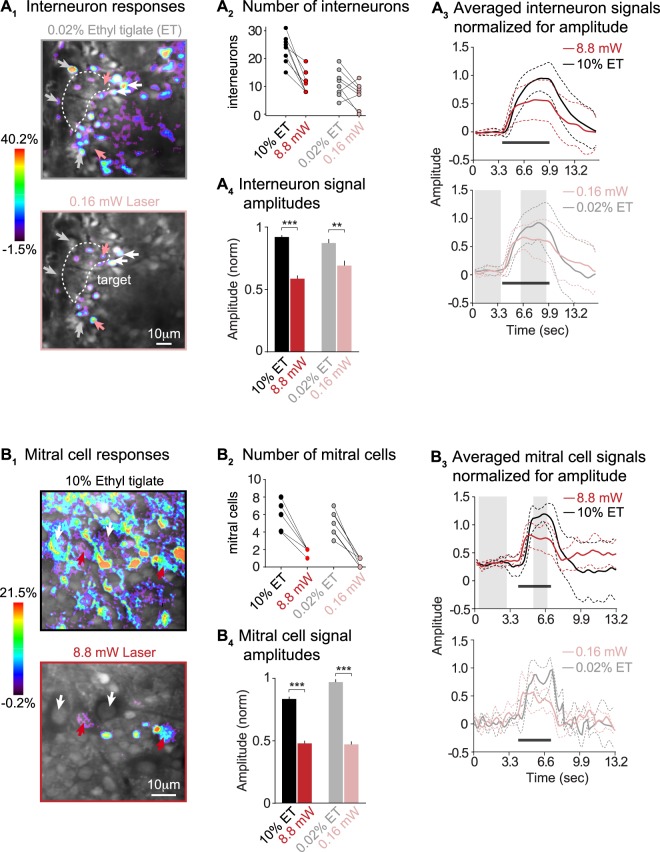
Reduced cellular activity surrounding a light-activated target glomerulus. (**A**_**1**_,**B**_**1**_) Frame subtraction images that compare the spatial distributions of ethyl tiglate (top) vs. laser-evoked (bottom) responses in (**A**_**1**_) glomerular layer interneurons and (**B**_**1**_) mitral cells. Glomeruli are indicated with dashed lines and some cellular responses are indicated with arrows. Frame subtractions were created by subtracting the imaging frames indicated with grey bars in **A**_**3**_ (bottom) and **B**_**3**_ (top). The scale bars in the bottom images of **A**_**1**_ and **B**_**1**_ also apply to the top images. (**A**_**2**_–**B**_**2**_) The numbers of ethyl tiglate vs. laser activated interneurons and mitral cells: each plot shows raw data for responding cells in a single imaging frame. (**A**_**3**_–**B**_**3**_) Averaged calcium signals from interneurons (**A**_**3**_) and mitral cells (**B**_**3**_). All data were recorded from cells that were located within a 30μm radius from the target glomerulus. The signals were normalized by dividing traces from matched odor-laser trials with the maximum fluorescence intensity during the evoked response at 4.6–9.2 sec. Dashed lines indicate standard deviations. (**A**_**4**_–**B**_**4**_) Histograms comparing signal amplitudes in the interneurons (**A**_**4**_) and mitral cells (**B**_**4**_): data were averaged and normalized as in **A**_**3**_–**B**_**3**_. The abbreviation ET denotes the odor ethyl tiglate.

Despite producing similar neuropil responses, ethyl tiglate vs. laser stimulation triggered visibly different activation of glomerular layer interneurons. Figure 3A_1_ shows interneurons that responded to ethyl tiglate vapor but not a laser stimulus (grey arrows) and to a laser stimulus but not to the vapor (pink arrows). The two cells that are indicated with a white arrow responded to both stimuli. Overall, fewer interneurons were activated during laser vs. ethyl tiglate stimulation (Fig. 3A_2_): only 56% of interneurons responding to 10% ethyl tiglate vapor also responded to a 8.8 mW laser stimulus, and only 68% of the cells activated by 0.02% ethyl tiglate vapor were also activated by 0.16 mW laser stimulation (4 glomeruli). Only a very small number of glomerular layer interneurons responded exclusively to laser stimuli: 3 of 206 and 11 of 191 in strong and weak laser vs. odor stimulation experiments, respectively. In addition, averaged peak calcium signals from glomerular layer interneurons were smaller when we compared the 8.8 mW laser vs. the 10% ethyl tiglate exposure condition (Fig. 3A_3-4_; t(148) = 9.11, p < 0.001, paired t-test), and the 0.16 mW laser vs. the 0.02% ethyl tiglate exposure conditions (Fig. 3A_3-4_; t(56) = 2.91, p = 0.0051, paired t-test).

Similarly, fewer mitral cells responded to laser vs. ethyl tiglate vapor stimuli. Fig. 3B_1_ shows a comparison between ethyl tiglate vapor (top) and laser-evoked (bottom) mitral cell responses. The cells indicated with the white arrow responded only to the odor vapor, while the cells indicated with the red arrow responded to both stimuli. Only 28% of the mitral cells that responded to 10% ethyl tiglate vapor also responded to 8.8 mW laser stimuli, and only 16% of the mitral cells that responded to 0.02% ethyl tiglate vapor also responded to 0.16 mW laser stimuli (Fig. 3B_2_; 5 glomeruli). We did not detect any mitral cells that responded to laser but not odor stimulation. Mitral cell signal amplitudes were also smaller when they were triggered by a 8.8 mW laser vs. 10% ethyl tiglate vapor (Fig. 3B_3-4_; t(9) = 20.18, p < 0.001, paired t-test) or a 0.16 mW laser vs. 0.02% ethyl tiglate vapor (Fig. 3B_3-4_; t(8) = 27.17, p < 0.001, paired t-test). Thus, laser stimulation of channelrhodopsin-2 expressing M72 olfactory neurons elicits fewer and smaller signals in both glomerular interneurons and mitral cells even though the sensory input to the target glomerulus was similar; this indicates that neuronal computations within the olfactory bulb contribute to the increased cellular activity associated with an odor stimulus.

### Temporal representation of laser stimuli in the glomerular unit

**Figure 4:**
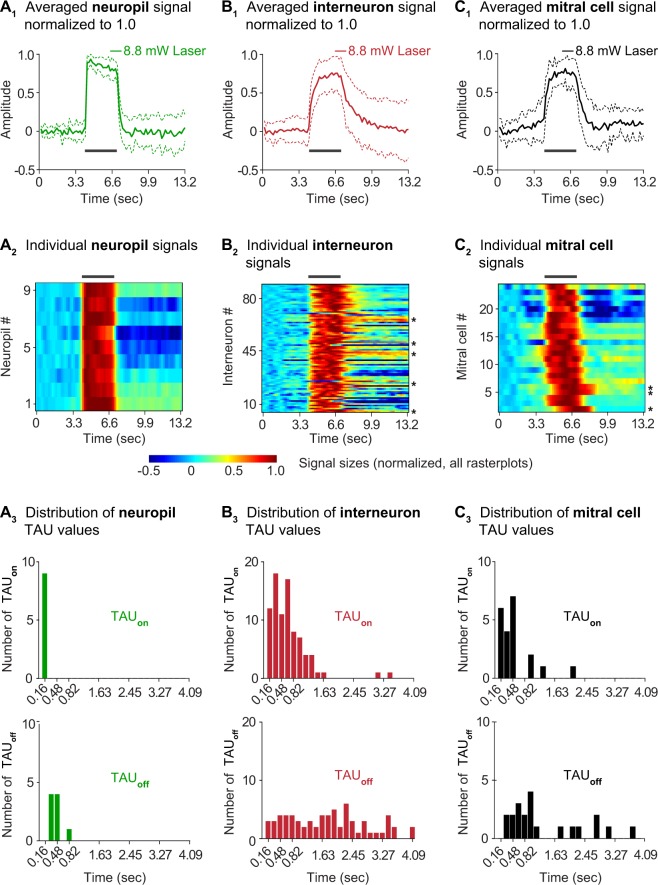
Temporal differences in calcium signals recorded from the three compartments of the light-activated target glomerulus. Averaged calcium signals from the neuropil (**A**_**1**_), the glomerular layer interneurons (**B**_**1**_), and the mitral cells (**C**_**1**_). The signals were normalized by setting the maximum of each trace to 1.0. To reveal their shapes, traces were not filtered. (**A**_**2**_,**B**_**2**_,**C**_**2**_) Raster plots showing raw signals from different glomerular compartments. The data were smoothed with 2 passes of a 1-2-1 binomial filter but not sorted. (**A**_**3**_,**B**_**3**_,**C**_**3**_) Frequency distributions of signal onset (TAU_on_, top) and signal offset (TAU_off_, bottom) times for signals from each of the three glomerular compartments. Neuropil TAU values bin within a few time points from the stimulus on- and offset. Interneuron TAU values are more diverse.

We next investigated temporal representations of optogenetic stimuli in all three compartments of the target glomerulus. The top panel of Fig. 4 shows averaged calcium signals from the neuropil (A_1_), interneurons (B_1_) and mitral cells (C_1_). These data were obtained in nine experiments in which all three compartments were imaged in the same animals (separate from the dataset above). All signals were normalized by setting the peak amplitude equal to 1.0 and left unfiltered to reveal the time course of each response type (as explained for Fig. 2E). Figure 4A_2_-C_2_ are raster plots that show the individual signals from each compartment. The signals are unsorted and appear in the order in which the experiments were conducted. Figure 4A_3_-C_3_ show frequency distributions of the signal onset (TAU_on_; top panel) and offset (TAU_off_; bottom panel) from each type of calcium signal.

The neuropil signal represented the laser stimulus with high temporal fidelity: TAU_on_ was very fast (164 msec) and binned neatly within one imaging frame after the stimulus delivery (Figure 4A_3_; the imaging frame rate for this experiment was 6.1 Hz, which is twice as fast as the frame rate used in Fig. 2E,F). The TAU_off_ was also rapid (455 +/− 52 msec) and the signal ceased almost immediately after stimulation (Fig. 4A_1–3_). Because of its speed and consistency, the neuropil calcium signal correlated very well to a square-shaped input stimulus (Euclidian distance correlation to square pulse = 0.94 +/− 0.005).

Signals from glomerular layer interneurons represented the laser stimulus to a lesser degree. The interneuron calcium signals rose continually during laser stimulation and often reached their maxima near the end of the stimulus delivery (Fig. 4B_1_). Interneuron calcium signals had a slower TAU_on_ than the neuropil (675 +/− 59 msec; t(92) = 2.84, p = 0.0055, unpaired t-test), and were more variable (Fig. 4B_3_). In many instances, interneuron signals outlasted the laser stimulus by several seconds (asterisks in Fig. 4B_2_). Overall, we calculated a relatively low correlation between the interneuron signal shape and a square pulsed laser stimulus (Euclidian distance correlation = 0.72 +/− 0.02).

Calcium signals from mitral cells represented the time course of the laser stimulus more reliably than signals from the interneurons. The mitral cell signals reached their peak mid-way through the stimulus delivery and returned to baseline shortly after the stimulus ended (Fig. 4C_1_). Only a few mitral cell signals outlasted the laser stimulation (asterisks in Fig. 4C_2_). The overall TAU_on_ for mitral cell signals was somewhat slower than that of the neuropil (485 +/− 99 msec; t(30) = 1.98, p = 0.056, unpaired t-test); the TAU_off_ was significantly slower than in the neuropil (1709 +/− 308 msec; t(30) = 2.51, p = 0.0175, unpaired t-test). The correlation of the mitral cell signal to a square pulse stimulus was 0.82 +/− 0.02. Thus, temporal differences are readily apparent in light-evoked signals across the glomerular unit. Stimulus representation occurs with high fidelity in the neuropil and the mitral cells, and to a lesser degree in glomerular layer interneurons.

### Spatial distributions of interneurons and mitral cells responding to light stimulation

#### Compact distributions of light-activated glomerular layer interneurons

**Figure 5:**
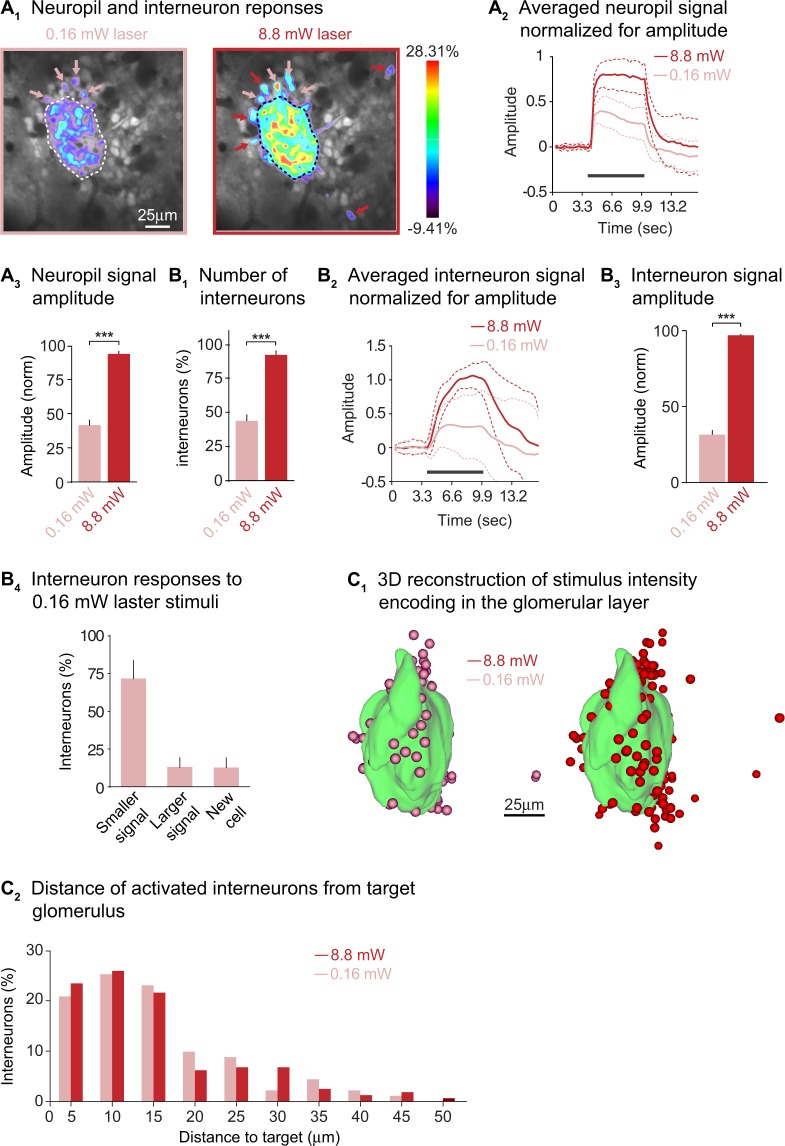
Compact distributions of light-activated glomerular layer interneurons. (**A**_**1**_) Frame subtractions showing a 7250 glomerulus activated with 0.16 mW vs. 8.8 mW laser stimuli. A stronger target signal and additional cell responses (red arrows) are visible in the 8.8 mW stimulation condition. The scale bar in the left panel applies to both images. (**A**_**2**_) Averaged calcium signals recorded from the neuropil during strong and weak laser stimulation. The signals were normalized by dividing each trace from pairs of strong laser and weak laser trials with the fluorescence signal during the response evoked by the strong laser stimulus. Dashed lines indicate standard deviations. (**A**_**3**_) The calcium signal amplitude in the neuropil of the target glomerulus was significantly larger in the 8.8 mW stimulus condition (normalized as in **A**_**2**_). (**B**_**1**_) More interneurons responded to 8.8 mW laser stimulation; the size of their responses also increased (**B**_**2–3**_). Calcium signals were averaged and normalized as described for **A**_**2**_. (**B**_**4**_) Analysis of interneurons that respond to 0.16 mW laser stimuli: the responses are organized based on what interneurons do in comparison to the 8.8 mW stimulus condition. (**C**_**1**_) Three-dimensional reconstructions of a 7250 glomerulus stimulated with 0.16 mW (pink, left panel) and 8.8 mW (red, right panel): more cells are recruited during strong stimulation and they cluster mainly near the target glomerulus (green). (**C**_**2**_) Frequency distribution showing the distances of interneuron responses from the border of the target glomerulus. Approximately 80% of cells are located within 20μm from the target for both laser stimulation intensities.

To examine the spatial distributions of light-activated glomerular layer interneurons, we imaged multiple single optical planes during stimulation with a 0.16 mW to 8.8 mW laser intensity. Figure 5A_1_ shows how the neuropil of the target glomerulus responded to these stimuli (dashed outline). An 8.8 mW laser stimulus produced a substantially larger neuropil calcium signal than a 0.16 mW stimulus (Fig. 5A_1-3_; t(14) = 12, p < 0.001, paired t-test). However, the time course of the neuropil calcium signals remained consistent (Fig. 5A_2_).

Glomerular layer interneurons responded to 8.8 mW vs. 0.16 mW stimulation with an almost two-fold increase in the number of responding cells. An example of this is shown in Fig. 5A_1_ where cells that responded only to 8.8 mW laser stimuli are indicated with red arrows, while cells that responded to 0.16 mW or both stimuli are indicated in pink. Figure 5B_1_ shows the overall population of interneurons responding to 8.8 mW vs. 0.16 mW laser stimuli, normalized against the maximum number of cell responses observed in each pairwise comparison (t(10) = 8.13, p < 0.001, paired t-test). The cellular calcium signals recorded during either 8.8 mW or 0.16 mW stimulation had similar time courses (Fig. 5B_2_), but their mean normalized amplitudes were substantially larger in the strong stimulus condition (Fig. 5B_3_; t(182) = 18.93, p < 0.001, paired t-test). We also analyzed the make-up of interneurons that responded to both stimuli; 72% responded with a smaller signal to 0.16 mW laser stimuli, 14% had a larger signal during 0.16 mW laser stimulation, while another 14% responded only to 0.16 mW laser stimuli (Fig. 5B_4_).

Figure 5C_1_ shows 3D reconstructions of a light-activated target glomerulus during stimulation with a 0.16 mW (left) vs. 8.8 mW (right) laser stimulus. The number of activated glomerular layer interneurons increases between the 0.16 mW and 8.8 mW stimulation conditions. However, the spatial distributions are similar, and the majority of the responding cells cluster near the glomerular neuropil. This is further illustrated in a histogram of the spatial distribution of responding cells during laser stimulation (Fig. 5C_2_). Seventy-eight percent of responding cells were located within 20μm of the target glomerulus during 0.16 mW laser stimulation; 77% during 8.8 mW laser stimulation. Hence there was no change in cell distributions (Fig. 5C_2_; p = 0.72, two-sample Kolmogorov-Smirnov test). The average distance of responding cells from the wall of the target glomerulus was 15 ± 2μm for 0.16 mW and 14 ± 1μm for 8.8 mW laser stimulation conditions (t(253) = 0.191, p = 0.847, unpaired t-test).

**Figure 6:**
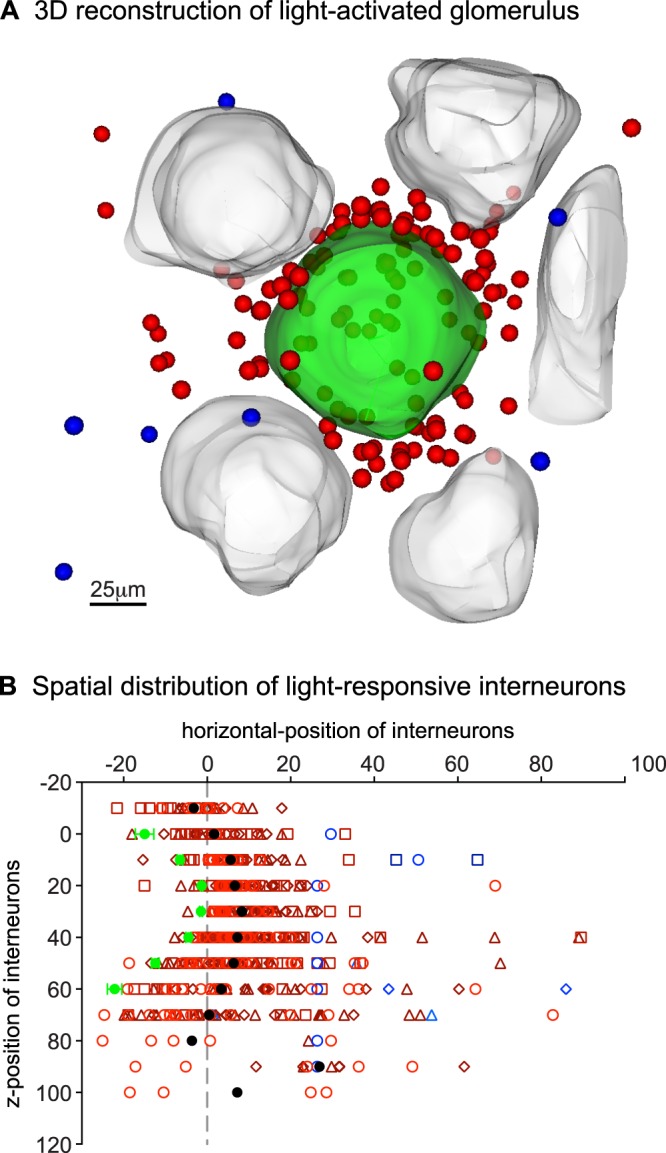
Average distribution of light-activated glomerular interneurons. (**A**) Three-dimensional reconstruction of the light-activated 7250 glomerulus in a 30-day old mouse. Light-activated cells (red) clustered in the interglomerular space separating the target glomerulus (green) from its non-responsive neighbors (grey). Inhibited cells (blue) are scattered throughout the imaging region. (**B**) Summarized locations of responding cells in four 30 day-old mice. The x-axis shows positions of the cells and an average glomerular border (solid green circles) with respect to a fixed width cylinder wall (dashed grey vertical line); the y-axis shows the normalized depth of the imaging plane binned to 10 μm intervals. The average glomerulus was 60 μm tall. The solid black circles represent the median distance of cell responses from the edge of the target glomerulus. Red symbols show activated cells while blue symbols show inhibited cells. Symbol shape and shading represent data from different animals.

We next mapped the average three-dimensional distribution of glomerular layer interneurons that responded to light stimulation in four postnatal day 30 mice. To do this, we repeatedly stimulated the glomerulus with 8.8 mW laser stimuli and recorded ensuing cellular responses through the depth of the glomerular layer while keeping the frame dimensions and x-y position constant. We recorded 116–157 cellular responses per target glomerulus. A reconstruction of a light-activated glomerulus is shown in Fig. 6A: the target glomerulus is green, responding cells are red and blue and neighboring glomeruli are grey. The similarity between Figs 5C_1_ and 6A is indicative of how consistent our findings were. The spatial distributions of 522 cells (from 4 glomeruli) responding to 8.8 mW laser stimuli are summarized in Fig. 6B. The black circles show the median locations of the responding cells (red symbols are individual cells). The contour of the target glomerulus is shown with green circles. Eighty-nine percent of activated cells were located within 30 μm from the border of the target glomerulus; only 3% were >50 μm from the target glomerulus. The average distance of cells with excitatory responses from the border of the target glomerulus was 16 ± 1 μm.

To ensure that our findings were representative of mice in all developmental stages, we reconstructed distributions of light-activated cells in mice aged 141–206 days. We recorded more cellular responses per glomerulus (Table 1), but the distributions of these cells remained unchanged. The average distance of the 686 light-activated cells from the border of the target glomerulus was 16.9 ± 0.6 μm, which is not different from the cell-to-glomerulus distance measured in 30 day old mice (see above).

**Table 1:** Number of light-responsive glomerular layer interneurons per target glomerulus in differently aged mice.

Mouse	Age (days)	Excitatory Responses	Inhibitory Responses	Stimulus intensity (mW)
1	30	114	2	8.8
2	30	125	3	8.8
3	30	127	7	16
4	30	156	2	16
5	141	230	2	8.8
6	181	208	6	8.8
7	206	248	9	16

#### Inhibitory responses in the glomerular layer

Using odor exposures, Homma *et al*. (2013) reported that 12% of interneurons connected to a glomerulus respond with inhibitory responses. We also observed inhibitory cellular responses in laser stimulation experiments, but only rarely (Table 1). Inhibitory cellular responses constituted 2.6% of the total responsive cell population in 30 day old mice. Inhibited cells were scattered around the target glomerulus, typically outside of the perimeter that contained most of the cells with excitatory responses (see blue cells in Fig. 6A). The positions of all inhibited cells in 30 day old mice are indicated with blue symbols in Fig. 6B. On average, these cells were located 54 ± 7 μm from the target glomerulus, significantly farther away than the average distance of cells with excitatory responses (16 ± 1 μm; t(535) = 13.2, p < 0.001, unpaired t-test).

#### Few laser-responsive cells are located outside the region containing the target glomerulus

**Figure 7:**
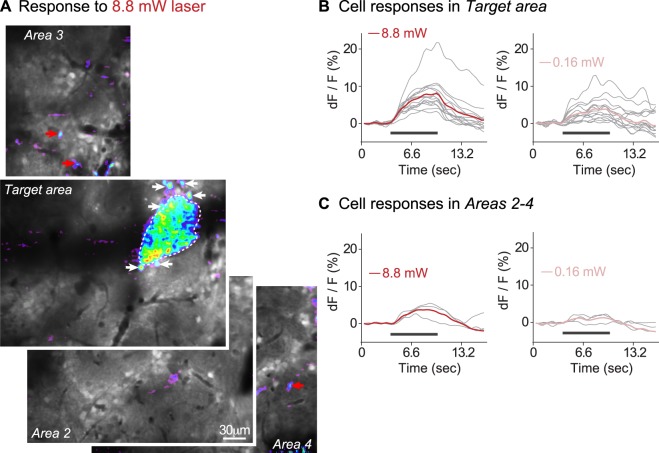
Laser-evoked glomerular layer interneuron responses in an extended field of view. (**A**) Frame-subtractions are overlaid on top of resting light images to show laser-evoked calcium signals. The target area contains the channelrhodopsin-2 expressing glomerulus, the remaining areas contain other glomeruli and were chosen based on image and labeling quality. The target area and areas 2–4 are 278 × 278 µm, area 3 is 278 × 125 µm. White arrows indicate cell responses around the target glomerulus (dashed outline); red arrows indicate cell responses outside the frame containing the target glomerulus. The scale bar in Area 2 applies to all four images. (**B**) Cellular responses within a 30 µm perimeter of the target glomerulus in response to 8.8 mW vs. 0.16 mW laser stimuli. (**C**) Cellular responses outside the target frame in response to 8.8 mW vs. 0.16 mW laser stimuli. Grey lines in (**B**,**C**) are individual cell responses; colored lines are their averages.

We searched for laser-evoked interneuron responses outside of the imaging frame containing the target glomerulus. Figure 7A shows the results from one preparation in which we imaged the region containing the target glomerulus, followed by three additional regions during 8.8 mW laser stimulation. We recorded 14 cell responses within a 30 µm radius from the target glomerulus. Several of these cells are indicated with white arrows in Fig. 7A and their calcium signals are shown in Fig. 7B. We detected calcium signals in an additional 3 cells outside a 30 µm radius from the target glomerulus, in frames that were not previously imaged (red arrows in Fig. 7A). The laser-evoked cellular responses had smaller amplitudes if they were located far away from the glomerulus (compare Fig. 7B,C). In total, we imaged 4580 μm^2^ of olfactory bulb tissue not containing the target glomerulus (five mice). In these experiments, we recorded 106 excitatory responses from cells that were located within 30 µm from the edge of the target glomerulus (per animal average = 21 ± 7 cell responses per single imaging plane). Outside of the frame containing the target glomerulus, we detected responses from another five cells (range 0–3 cell responses in 5 mice), which corresponds to just 4.7% of the total responding cell population.

To summarize, the spatial distribution of light-activated glomerular layer interneurons is very compact, even during strong laser stimulation. Stimulus intensity is encoded primarily via the recruitment of new cells inside a restricted cluster.

#### Distributions of light-activated mitral cells

**Figure 8:**
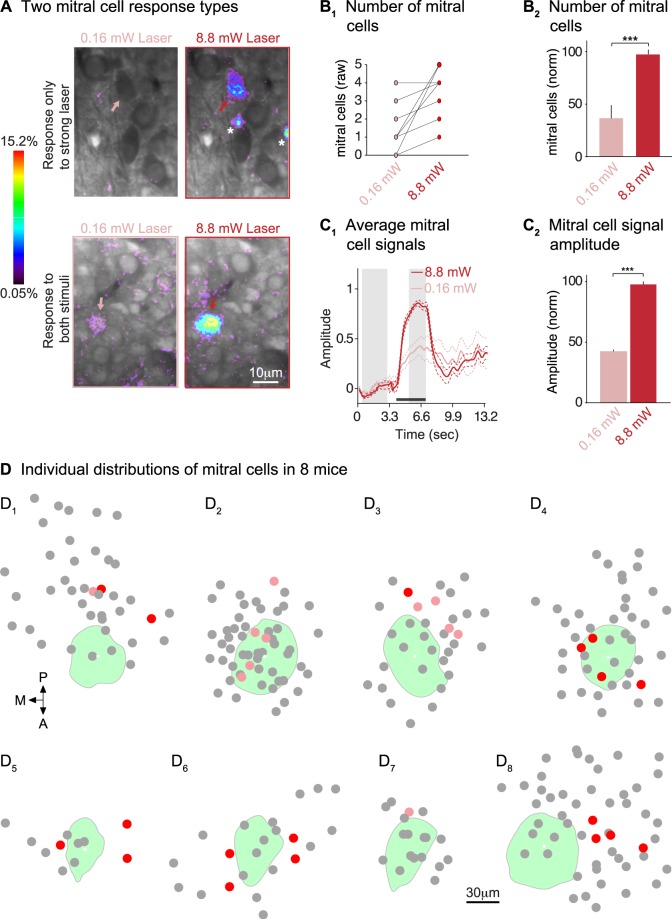
Compact distributions of light-activated mitral cells. (**A**) Frame subtractions showing two distinct mitral cell signals in response to 0.16 mW vs. 8.8 mW laser stimuli. The top panel shows a cell that responds only to 8.8 mW laser stimuli (arrows). The bottom panel shows a cell that responded to both stimuli (arrows). The scale bar on the bottom right applies to all panels. (**B**_**1**_) The number of mitral cell responses often increased with stronger laser stimuli; shown are data from individual imaging frames. (**B**_2_) Significantly more mitral cells responded to 8.8 mWvs. 0.16 mW laser stimulation (9 mice). (**C**_**1**_) Averaged calcium signals recorded from mitral cells during strong and weak stimulation; signals were normalized by dividing traces from matched strong laser vs. weak laser trials with the mean fluorescence intensity during the response by the strong laser stimulus. Dashed lines indicate standard deviations. (**C**_**2**_) Mitral cell calcium signals were significantly larger in the 8.8 mW vs. 0.16 mW stimulus condition (normalized as in **C**_**1**_). (**D**) Planar distribution of mitral cells around the target glomerulus. Glomeruli are shown in green; mitral cells that responded only to 8.8 mW stimuli are pink dots, while cells that responded to 0.16 mW and 8.8 mW stimuli are red dots. Grey dots are mitral cells that were visible and had an odor response but lacked a laser response. The scale bar applies to all samples.

We studied the numbers and distributions of light-activated (and inactive) mitral cells in eight glomeruli. We first imaged mitral cell responses directly under the target glomerulus, within a consistent x-y position, through a depth of 20–40 μm. This covered the thickness of the mitral cell layer. Additional imaging regions were then surveyed by extending the imaging region in a quasi-circular pattern around the frame containing the target glomerulus. The mitral cells responded to 0.16 mW vs. strong 8.8 mW laser stimuli with distinct activation patterns and/or graded signal sizes. The top panels in Fig. 8A show a mitral cell that did not respond to 0.16 mW stimulation (pink arrow) but did respond to 8.8 mW laser stimuli (red arrow). The two smaller hotspots of activity in the top right panel of Fig. 8A (asterisks) presumably represent the activity of granule interneurons. The bottom panel of Fig. 8A shows an example of a mitral cell that responded to both 0.16 mW and 8.8 mW laser stimuli (see pink and red arrows), albeit with different signal sizes. The minimum number of light-activated mitral cells was zero per glomerulus and the maximum number was five per glomerulus (Fig. 8B_1_; n = 9 glomeruli). On average we recorded 3.5 ± 1.5 mitral cell responses per preparation, approximately twice as many responded to 8.8 mW vs. 0.16 mW laser stimuli (t(8) = 5.06, p < 0.001, paired t-test; Fig. 8B_2_). Mitral cells responded to 0.16 mW and 8.8 mW laser stimuli with similarly shaped signals (Fig. 8C_1_; these are separate from those shown in Fig. 3B_2_). The mean normalized amplitudes of these signals were much larger during 8.8 mW vs. 0.16 mW laser stimulation conditions (Fig. 8C_2_; t(26) = 26.31, p < 0.001, paired t-test). All mitral cell signals were either smaller or absent in the 0.16 mW laser stimulus condition.

The distributions of light-activated mitral cells are summarized in Fig. 8D. These plots show the positions of all mitral cells, regardless of their responsiveness. The result is a sparse responding pool of mitral cells: red circles show cells that responded to 0.16 mW and 8.8 mW laser stimuli, while pink circles show cells that responded only to 8.8 mW stimuli. Grey circles indicate mitral cells that had odor responses but lacked a response to laser stimulation. Mitral cells that responded to laser stimuli, despite being sparse, clustered preferentially near each other. The average distance between responding cells was 34 ± 2 μm, which was smaller than the average distance between all other mitral cells (47 ± 2 μm; t(3785) = 3.24, p = 0.0012, unpaired t-test). Interestingly, only 7 out of the 29 responding mitral cells, in 2 out of 8 specimens, were located directly underneath the target glomerulus (Fig. 8D_2_,D_4_). The remaining cells were located in a lateral or posterolateral direction, at a mean distance of 41 ± 5 μm from the center of the glomerulus (average calculated from all cells). To summarize, mitral cell responses to optogenetic stimulation are very sparse. Small clusters of mitral cells that respond to laser stimuli are often located nearby the target glomerulus, rather than directly underneath it.

## Discussion

Odors evoke widespread olfactory bulb activity, which complicates the study of individual glomeruli. Some labs have overcome this problem via direct optogenetic activation of individual glomeruli to study olfactory bulb outputs^12,31^ and their relationship to olfactory behaviors^14^. We used an optogenetic strategy to study the neuropil (input), interneuron (modulatory) and mitral cell (output) activities related to single glomerular activation *in vivo*. The light-activated glomerulus has a compactly organized cellular compartment and provides a simplified *in vivo* model to study input-output transformations in the olfactory bulb.

### Technical considerations

#### All optical stimulation and recording of neuronal activity

To our knowledge, laser stimulation together with simultaneous optical imaging is only rarely attempted. The obvious challenge is that the combination of laser stimulation with sensitive optical recording equipment causes optical artifacts (Fig. 1D). We overcame this problem by coupling the laser stimulus delivery to the imaging scan cycle. Our method constrains the allowed stimulation frequency to a multiple of the scanning speed. With regard to the olfactory system, the laser stimulation frequencies of 3–6 Hz adequately cover natural breathing rates; anesthetized mice breathe at 3–4 Hz^32^.

#### Optical stimulation of sensory neurons to elicit activity in a glomerulus

We stimulated channelrhodopsin-2 expressing sensory neurons to elicit neuronal activity in a pair of upstream olfactory bulb glomeruli (Fig. 1C). Other laboratories have directly stimulated the glomerular neuropil to elicit optogenetic activity in mitral cells^12,31^ or to elicit olfactory behaviors^14^. Our laser stimulus was a divergent beam in a highly scattering tissue, and it presumably provided light stimulation of most, if not all channelrhodopsin-2 expressing sensory neurons. Increasing the laser stimulus to an average intensity of 8.8 to 16 mW did not increase the size of the glomerular response (Supplemental Fig. 1A) and we conclude that our method produced optimal activation conditions for the channelrhodopsin-2 expressing glomerulus. Nevertheless, a possible confound of our method exists in the innate responsiveness of glomeruli to multiple odor stimuli. Glomeruli almost always respond to multiple odors, and few or no glomeruli are truly selective. Hence, glomeruli are not generally active in isolation and it is not a straightforward task to compare calcium signals in a glomerulus that is activated selectively to calcium signals that are triggered with odor stimuli. Competing or facilitating interactions with neighboring glomeruli could affect the amplitude(s) of the glomerular response in the case of odor stimuli. Additional experiments are required to understand this aspect of glomerular excitability.

#### Neural vs. glial calcium signals

We did not discriminate between calcium signals from neurons vs. glia. Astrocytes respond to odor stimuli^33^, but their signals are smaller than those of neurons, and are only detectable in a small proportion glomerular layer cells^30^. We therefore assume that activity in astrocytes accounted for only a small fraction of the responding cells.

### Odor vs. light-evoked calcium signals in the target glomerular unit

#### Consistent odor and laser-evoked neuropil responses

Our strongest odor stimulus was 10% ethyl tiglate vapor. This exceeds the odor concentrations used in other experiments^10,20,29,34,35^ and presumably produces ‘maximum’ stimulation of the olfactory sensory input. In contrast, the weak odor and laser stimuli represent more natural olfactory inputs. We were able to replicate the shapes and amplitudes of both strong and weak odor-evoked neuropil responses using strong and weak laser stimuli (see Fig. 2C), and conclude that we mimicked the naturally occurring sensory input to a single glomerulus.

#### Light vs. odor activation of glomerular interneurons

Homma *et al*.^30^, describe a glomerular unit as a principal glomerulus and a shell of juxtaglomerular interneurons with the same odorant responsiveness. Eighty percent of the interneurons were located within 20 µm from the glomerulus. Based on this result, we focused comparisons between odor and laser-evoked cellular responses to 30 µm around the target glomerulus, and found that ~35% fewer glomerular layer interneurons responded to laser vs. odor stimuli (see Fig. 3A_2_). This finding illustrates the difficulty of defining a functional glomerular unit based on odor-exposures alone. Neighboring glomeruli are separated by narrow spaces that contain hundreds of interneurons^36^. Many of these neurons are shared or not connected to the presumed target glomerulus. The reduced number of laser vs. odor-responsive cells may thus imply that we activated only the cells that are connected to the 7250 target glomerulus.

Another possible explanation relates to the type(s) of interneuron that responded to channelrhodopsin stimulation. Glomerular layer interneurons are either uniglomerular or multiglomerular, and may partake in the activation/inhibition of one or several glomeruli. Forty percent of glomerular layer interneurons are multiglomerular^15^ and, hence, may require input from multiple glomeruli to create sufficient activity to be detected by calcium measurements. This could explain why we observe a 35% decrease in the number of interneurons activated by optogenetic stimulation.

#### Estimating the population of glomerular layer interneurons

Parrish-Aungst *et al*.^36^, estimated that a glomerular unit in mice includes ~680 cells, of which 440 are glomerular layer neurons. These anatomical data do not specify functional connections between interneurons and their putative parent glomeruli. We counted between 114 responding interneurons in 30 day old mice to 248 interneurons in >8 week old mice (Table 1). These estimates are clearly smaller than anatomical cell counts. Additional neurons may have been activated less strongly by laser stimulation, so that their optical signals were below the noise threshold of our measurements. Furthermore, cells with inhibited signals may have been overlooked. Homma *et al*. (2013) reported that 12% of glomerular layer interneurons are inhibited by odor exposures. This inhibition presumably occurred because of lateral interactions between co-activated glomeruli, which would not have occurred in our experiment. Furthermore, inhibition is difficult to detect using calcium imaging except in neurons with elevated calcium from spontaneous activity^30^.

#### A sparse light-activated mitral cell pool

Several dozen mitral/tufted cells project their primary dendritic tufts into the neuropil of one glomerulus^12,37,38^. Mitral cells are activated by monosynaptic and polysynaptic inputs from the sensory neurons, external tufted cells^18,39,40^, and periglomerular interneurons^41^. Within a glomerulus, mitral cells are connected to each other via a lateral excitatory network, which enhances their excitability and boosts glomerular output^42,43^. Therefore, one might presume that mitral cells are highly sensitive to odor and/or light stimulation.

Surprisingly, we recorded from maximally five mitral cells per light-activated target glomerulus, even during high intensity laser stimulation. Dhawale *et al*.^12^, reported signals from approximately four mitral cells per selectively activated glomerulus. Why is the number of responding mitral cells so low? Since we activate only one glomerulus, we likely reduced lateral excitatory mechanisms that could facilitate mitral cell activation. Glutamate spillover results in interglomerular mitral cell co-activation^42^. Perhaps certain mitral cells (i.e., those that we failed to activate) are wired to detect coincidental activation from multiple glomeruli. Considering that a glomerulus is rarely activated in isolation by odorants it would make sense that some mitral cells are also tuned to detect activity outside of their parent glomerulus.

### Imaging olfactory bulb activity in anesthetized mice

All of our recordings were obtained from mice that were deeply anesthetized, and it is important to consider the potential effects of anesthesia on neuronal activity in the olfactory bulb. Blauvelt *et al*.^44^, reported stable spatial activation patterns of mitral cells in anesthetized vs. awake states^44^. Kato *et al*.^45^, reported a sparsening in the number of responding mitral cells in awake mice^45^, and Wachowiak *et al*.^35^, showed that interneuron and mitral cell activation patterns closely reflected glomerular input in anesthetized mice, while more diffuse and complex activation patterns accompanied awake states^35^. We therefore conclude that the low numbers of responding interneurons and mitral cells observed during optogenetic stimulation cannot be attributed to the effects of anesthesia.

Aside from changes in spatial activation patterns, it has been consistently reported that interneuron and mitral cell responses become more dynamic and complex in awake versus anesthetized animals^35,45^. This indicates that olfactory information processing involves temporal coding. Our imaging data were acquired with a relatively slow frame rate (3–6 Hz), which precluded us from identifying small temporal changes in cellular activation. Hence, we can describe consistencies in the spatial activation of cellular activation, but we have yet to determine how optogenetic activation of a single glomerulus affects temporal features in signals recorded from different types of olfactory bulb neurons.

### Variable stimulus representation in glomerular layer interneurons

The temporal features of the laser stimuli were represented with high fidelity in signals from the glomerular neuropil and with somewhat less fidelity in mitral cells (compare Fig. 4A_1_–C_1_). The temporal information carried by the external stimulus is thus preserved in glomerular input-output transformations. However, the stimulus time course is less accurately reflected in glomerular interneuron responses, which highlights an important, but often overlooked, finding related to the functional diversity of the interneurons. Up to 20 molecularly distinct interneuron types have been recognized^15,36,46^, and several different functional roles have been attributed to certain subtypes^15,18,22,28,47^. Yet, recent calcium imaging studies on glomerular layer interneurons reported primarily similarities in their odorant responsiveness^30,34,35^, while activity differences were rarely mentioned. Our results in Fig. 4B_1-3_ point to the diversity of the interneuron population response. Going forward, it will be important to investigate functional differences among interneurons in more detail.

### Eliciting olfactory behavior

Smear *et al*.^14^, used optogenetic stimulation to activate a single glomerulus and successfully trained mice to respond behaviorally to this selective glomerular activation. Our data complement this experiment and suggest that these olfactory behaviors may have originated from the activity of extremely few mitral cells, 2–5 to be precise. This alone is remarkable, especially if we consider how complex the odor-elicited glomerular activation is. Identification of downstream targets of these mitral cells will still be difficult, given that individual mitral cells project axons to very large regions of the brain^13^. However, our method simplifies this feat and opens the way to more targeted studies of the brain regions involved in generating olfactory behaviors.

## Materials and Methods

### Animal use

All animal experiments were performed in accordance with the protocols approved by Yale (IACUC Protocol # 2015-10196) and the Korea Institute of Science and Technology (IACUC Protocol #2015-003).

Fifty-four mice of both sexes aged 1–12 months were used. The number of mice in each experiment is listed in the results. An additional 25 mice were used to establish imaging and optogenetic stimulation protocols.

### Transgenic mice

Gene-targeted mice (*M72* → *S50-IRES-hChR-Venus;* hereafter called 7250 mice) were created by replacing the coding sequence at the native S50 olfactory receptor from the start to the stop codon with that of the olfactory receptor M72 (Fig. 1A). An internal ribosome entry site (*IRES*) was inserted after the M72 coding sequence followed by the gene for codon optimized (humanized) channelrhodopsin-2 (H134R) fused to Venus YFP (hChR2V). To do this, an *IRES-hChR2-Venus* AscI cassette was cloned into the M72 → S50 targeting vector^48^. The targeting vector was electroporated into embryonic stem cells (129S6 strain) and clones were screened for homologous recombination by long-range PCR. Chimeras were generated by morula aggregation using C57BL/6 embryos. Transgenic 7250 mice expressed channelrhodopsin-2 venus YFP in olfactory sensory neurons in the dorso-posterior olfactory epithelium and in symmetric pairs of glomeruli on the medial and dorsal olfactory bulb surfaces (Fig. 1B). Recordings were obtained from dorsal glomeruli.

### Animal preparation and surgery

Anesthesia was induced by an intraperitoneal injection of Ketamine/Xylazine (80/8 μg/g of body weight) and monitored by toe pinch reflex and observation of whisker movements. Additional Ketamine/Xylazine (40/4 μg/g of body weight) was injected to maintain anesthesia. Recordings started five minutes after anesthetic delivery. Respiration was monitored for consistency and the body temperature was kept at 36–37 °C.

Prior to surgery, 0.05 mL dexamethasone (0.5%) and 0.05 mL bupivacaine (0.5%) were administered via intramuscular injections into the animals’ back and subcutaneously between the ears, respectively. The skin covering the skull was removed from behind the ears to just posterior to the nose. A headpost was attached to the cleaned skull using cyanoacrylic glue and dental cement (Orthoplast, Vertex Dental, Singapore). The location of the channelrhodopsin-2 venus YFP glomerulus was determined using a Leica M165 stereomicroscope equipped with fluorescence optics. The skull above the glomerulus was thinned using a high-speed dental drill (Foredom K.1070) and bilateral craniotomies were performed (d = 1.5 mm × 1.5 mm). The bone overlying the olfactory epithelia was also thinned to permit laser stimulation of the channelrhodopsin-2 expressing olfactory receptor neurons.

### Multi-cell bolus-loading and imaging windows

We used the multi-cell bolus-loading technique^49^ to label the olfactory bulbs. Fura PE-3 AM (Teflabs, Austin, TX) or OGB-1 BAPTA AM (ThermoFisher, Waltham, MA) were freshly dissolved in 20% Pluronic F-127 in DMSO (ThemoFisher), and diluted to 0.05 mM in 2.5% Fast Green (Sigma, St. Louis, MO) in a solution containing in mM: 150 NaCl, 2.5 KCl and 10 HEPES, pH 7.4 (final Pluronic F-127 concentration ≈ 0.5%). After sonication and filtering (0.45 µm Ultrafree centrifugal filter, Millipore, Billerica, MA), the dye solution was injected into the olfactory bulbs through a broken back glass microelectrode with a tip diameter of ~12 µm using a Nanoliter Injector (World Precision Instruments, Sarasota, FL). Approximately 1 µl dye solution was injected around the edge of the craniotomy. The olfactory bulbs were then covered with 2% agarose and a coverglass. Another window was installed over the olfactory epithelia (Fig. 1C).

Laser stimulation caused significant optical interference with simultaneous two-photon imaging (see below). To minimize this interference, we covered all areas that were exposed during surgery (except windows) with black dental acrylic. A black cardboard screen was also installed between the olfactory epithelium and the olfactory bulbs to block the stimulation laser light from reaching the microscope objective (Fig. 1C).

### Two-photon imaging

Optical imaging was performed with a custom two-photon laser-scanning microscope (Sutter, Novato, CA). Two-photon illumination was provided by a Chameleon Vision S mode-locked Ti-sapphire laser (Coherent, Glasgow, UK) and was controlled with a Conoptics 350-80 Pockel’s Cell and a Uniblitz TS6B Shutter (Vincent Associates, Rochester, NY). Laser scanning was performed with an XY pair of 3 mm dielectric coated mirrors mounted on 6215 galvo motors (Cambridge Technologies, Bedford, MA). The microscope was equipped with a fixed lens mount to simplify the optical path; a Nikon CFI 25× 1.10 N.A. or a Nikon CFI 16× 0.8 N.A. lens were used for all experiments. Positioning of the mouse relative to the objective was performed with an XYZ movable stage, composed of an Aerotech AVS 125 vertical translator (Pittsburgh, PA) and a Sutter MT78 motorized large XY platform stage.

The 7250 target glomerulus was visualized using 920–960 nm excitation; Fura-PE-3 was excited at 800 nm, OGB-1 was excited at 920 nm. Emitted light was passed through a 510/84 bandpass filter (Semrock, Rochester, NY) and collected with a GaAsP photomultiplier (H10770, Hamamatsu, Hamamatsu City, Japan). Regions of interest were scanned at 3.05 or 6.1 frames per second. All optical components were controlled with Sutter Mscan.

### Odor delivery

Odor stimulation was controlled with a custom-built olfactometer^25^. Ethyl tiglate (Sigma), a ligand for the 7250 glomerulus^50^, was used at 0.02% to 10% of saturated vapor for all odor experiments. Odor stimuli were delivered as 2.8 to 5 sec pulses.

### Laser stimulation of channelrhodopsin-2 expressing sensory neurons

Laser stimulation of channelrhodopsin-2 expressing olfactory sensory neurons was performed with a MLL-FN 447 nm laser (CNI Laser, Changchun, China), delivered via a 25 µm fiber-optic cable (Thorlabs, Newton, NJ) onto the olfactory epithelium window as a ~1.5 mm spot (Fig. 1C). The laser stimulation power was checked daily and is provided as the average laser stimulation intensity (e.g., a 1 mW stimulus delivered as 60 ms pulses at 3 Hz resulted in an 18% duty cycle which equals 0.18 mW average stimulation intensity).

Laser stimulation produced visible optical interference with simultaneous 2-photon imaging (Fig. 1D). We initially tried to eliminate this artifact by blocking the blue stimulation laser light from reaching our photomultipliers with bandpass or edge filters placed in front of the photomultiplier tubes. However, even after the installation of multiple filters in series we could still detect the stimulation artifact in our data. We therefore timed the laser stimulation so that the epithelium was only illuminated when the vertical scan mirror was at the start or return position of the imaging scan cycle. This limited the optical interference to the top and bottom edges of the scanned image, and excluded laser interference from central regions (Fig. 1D,E). The following stimulation patterns were used: (1) a train of 60 ms laser pulses separated by 305 ms for a 3.0 Hz galvo scan at 512 × 512 resolution; (2) a train of 40 ms laser pulses separated by 120 ms for a 6.1 Hz galvo scan at 300 × 300 resolution. Laser pulses were delivered either as single pulses or as repeating 2 ms segments of laser ON and OFF (40 to 60 ms total); the signals evoked by these stimuli were the same.

Laser stimulation powers were determined by exposing channelrhodopsin-2 expressing olfactory-receptor neurons to a range of laser intensities. Responses were detected for stimulation intensities ranging from 0.012 to 16 mW. Stimulation with a 0.16 mW laser produced the most consistent recordings for the ‘low power’ condition but lower intensities were used when signals were visible (Supplemental Fig. 1A). The maximum stimulation intensity was capped at 16 mW because stronger stimulation intensities elicited non-optogenetic calcium signals in wild type mice (Supplemental Fig. 1B). There were no detectable signals in non-stimulus trials.

### Image and statistical analyses

#### Representation of calcium signals and analysis

Calcium imaging data were analyzed with NeuroPlex (Redshirt Imaging, Decatur, GA), FIJI (https://fiji.sc/), and Matlab (Natick, MA). Signals were initially identified with the NeuroPlex frame subtraction procedure. In this approach, 5–10 frames preceding the stimulus presentation were subtracted from a similar number of frames during the stimulus presentation. The resulting frame subtractions indicated pixels with a fluorescence intensity change (e.g., Fig. 2A top left). Additional regions of interest were manually inspected to ensure inclusion of small signals. Analyses were conducted independently by 2–3 experimenters. All frame subtraction images were smoothed using 2–3 repetitions of a low pass 3 × 3 pixel kernel filter with the center pixel weighted at 3.

All calcium signal traces are shown as fractional fluorescence changes, ∆F/F, which were obtained by dividing the fluorescence intensity change in the region of interest by the fluorescence intensity recorded prior to the stimulus delivery. The calcium signal traces are single trial recordings in Fig. 1E and two trial averages in Fig. 7B,C and Supplemental Fig. 1B_2_. Calcium signal traces in Figs 2C, 3A_3_,B_3_ and 5A_2_,B_2_ were averaged across multiple experiments and were normalized by dividing each trace by the maximum fluorescence change that was recorded in pairwise stimulation conditions (odor vs. laser) or stimulus intensities (strong vs. weak laser). The maximum fluorescence was defined as the mean fluorescence intensity during the evoked response. All traces were low-pass filtered with 2 to 4 passes of a 1-2-1 binomial filter in NeuroPlex or Matlab, and amplitude differences for statistical comparisons were obtained from 3 frames (maximum intensity + two adjacent) from each normalized and smoothed trace. Calcium signal traces in Figs 2E and 4A_1_–C_1_ are averaged across multiple experiments and normalized by setting the maximum of each trace to 1.0. The traces were not low-pass filtered in order to preserve temporal features. We defined the rise time, TAU_on_, of each trace, as the time it took for the signal to rise to 63% of its maximum intensity following the onset of stimulation. TAU_off_ was defined as the time it took for the signal to decay by 63% after offset of stimulation.

#### Two-dimensional morphological analysis

Two-dimensional morphological analyses were conducted to determine the location of cellular responses around the glomerulus within a single imaging plane (i.e., the mitral cell layer in Fig. 8D). Imaging frames were assembled into a collage based on their XY coordinates in Illustrator (Adobe, San Jose, CA). The centroid of the target glomerulus was identified and XY positions of individual cells were used to calculate their Euclidian distance from this centroid and from each other.

#### Three-dimensional morphological analyses

Three-dimensional reconstructions of glomeruli and interneurons were created with TrakEM2 Software^51^. The glomerular neuropil outline was manually traced in 2 μm spaced optical sections. The positions of responding cells were identified in individual z sections. The distributions of odor or laser-responsive cells around the target glomerulus were summarized in X-Y plots (e.g., Fig. 6B). To compress the results from multiple target glomeruli of non-identical shapes and sizes onto a single plot, we first fitted each target glomerulus with a cylinder that extended through its depth. The diameter of this cylinder was equal to the maximum width of the glomerulus. The location of the glomerular border (Fig. 6B, green dots) was determined for each optical section in which the glomerular neuropil was visible by measuring the distance between the cylinder wall and the glomerular border. We then used TrakEM2 to measure the planar distances of each responding cell centroid from the wall of the cylinder in each z plane. The resulting planar distances (x) were then normalized by scaling to a cylinder with a diameter equal to the average maximum diameter from the included glomeruli. The vertical positions of the cells (y in Fig. 6B) were fitted onto a common z-axis. To do this, we determined the height of each glomerular neuropil from raw data and calculated the height of the average glomerulus. Individual z-sections were then scaled to compress/stretch vertical dimensions of individual glomeruli; scaled values were binned into 10 μm sections and the positional information of cells and glomeruli was used to calculate the planar distance between cells and the average target glomerulus.

## Electronic supplementary material

Figure S1
