# Supplementary material for: Sparsened neuronal activity in an optogenetically activated olfactory glomerulus
Source: Sci Rep. 2018 Oct 8;8:14955. doi: 10.1038/s41598-018-33021-w (PMC6175855; doi:10.1038/s41598-018-33021-w)
Supplement: Supplementary file 1 — Figure S1 [file 41598_2018_33021_MOESM1_ESM.pdf]

Supplementary Material

Sparsened neuronal activity in an optogenetically activated olfactory glomerulus.

Oliver Braubach[1,2,3,4], Tuce Tombaz[1,2,3], Tristan Geiller[1], Ryota Homma[2,3], Thomas Bozza[5,6], Lawrence B. Cohen[1,2,3], Yunsook Choi[1,2,3].

[1] Center for Functional Connectomics, Brain Science Institute, Korea Institute of Science and Technology (KIST),
Seoul, 136-791, Korea.
[2] Department of Cellular and Molecular Physiology, Yale University School of Medicine, New Haven, CT, 06510,
USA.
[3] NeuroImaging Cluster, Marine Biological Laboratory, Woods Hole, MA, 02543, USA.
[4] Department of Neurosurgery, Cedars Sinai Medical Center, Los Angeles, CA, 90048, USA.
[5] Department of Neurobiology, Northwestern University, Evanston, IL, 60208, USA.
[6] Visiting Scientist Program, HHMI Janelia Farm Research Campus, Ashburn VA, 20147, USA.

A Calcium signal amplitude in neuropil and number of interneuron reponses

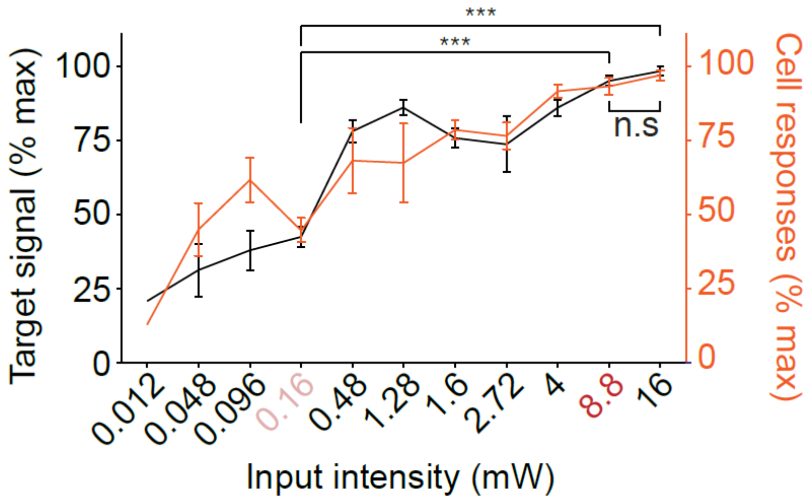

B_1 Non-optogenetic signals in wild-type mice: frame subtractions

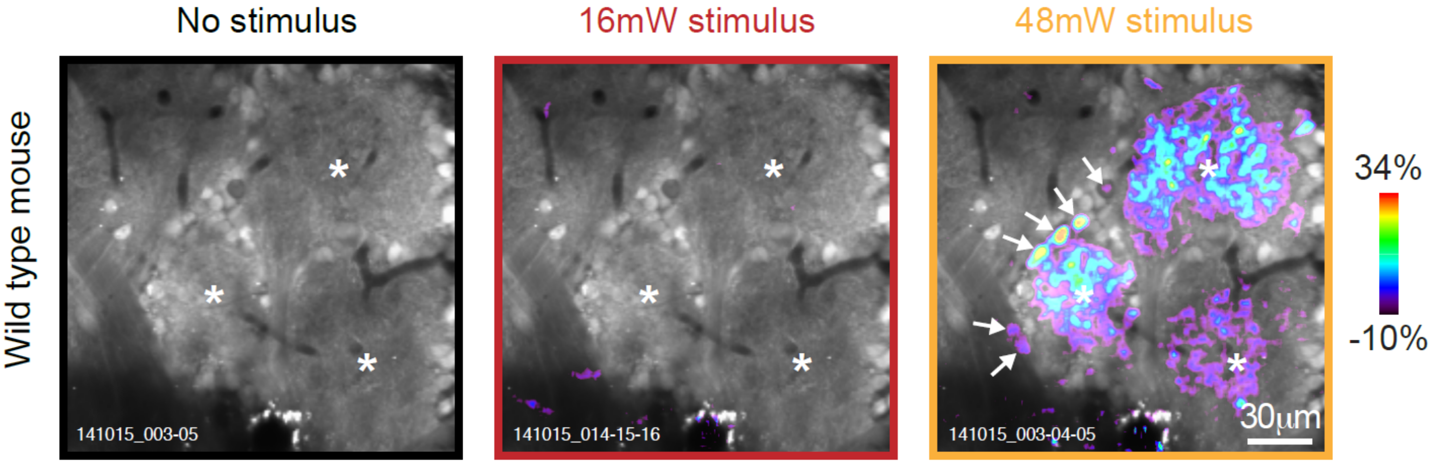

B_2 Non-optogenetic signals in wild-type mice: intensity vs. time traces

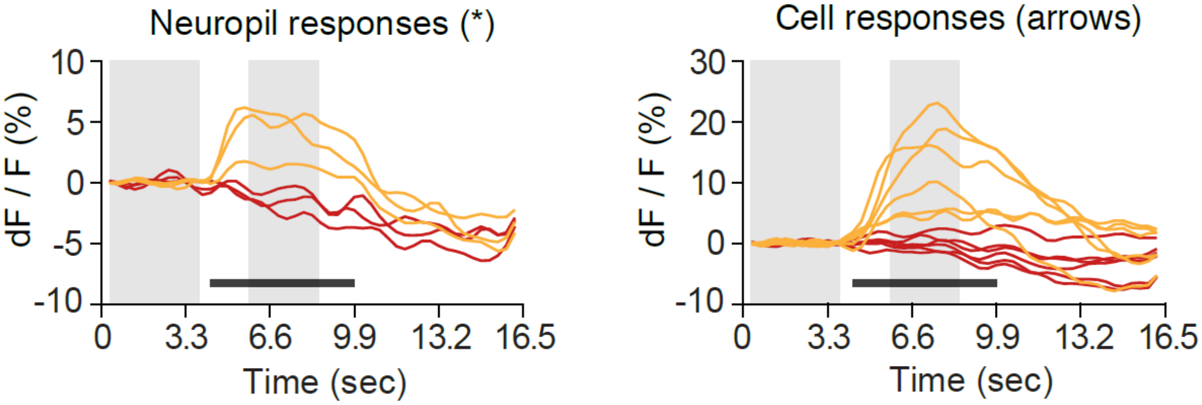

Supplemental Figure 1: Determining minimum and maximum laser stimulation intensities. Experiments in (**A**) were conducted with channelrhodopsin mice, while experiments in (**B**$_{1\text{-}2}$) were performed with wild type (WT) mice. (**A**) Plot showing the signal amplitude recorded from the target glomerulus (black) and the numbers of activated glomerular layer interneurons (orange) as a function of laser stimulation intensity. Data were normalized by dividing each signal or number of responding cells by the corresponding maximum that was observed in a stimulation intensity series. The weakest stimulation intensity that produced measurable calcium signals was 0.012 mW, but signals were inconsistent and only observed in 1/5 experiments. A 0.16 mW laser stimulus produced more consistent recordings (responses in = 9/10 mice) and was generally chosen for the ‘weak power’ condition. The maximum ‘safe’ stimulation intensity was determined to be 8.8 mW to 16 mW; no difference was found in the amplitude of glomerular signals evoked by 8.8 mW or 16 mW ($p = 0.0836$; paired t-tests). (**B**) The maximum ‘safe’ stimulation power was set at 8.8 - 16 mW because higher power stimuli elicited non-optogenetic calcium signals in wild type mice. (**B**$_1$; left) Fura-labeled imaging region: three glomeruli are indicated with asterisks. (**B**$_1$; middle) Frame subtraction for an experimental trial in which the olfactory epithelium was stimulated with a 16 mW average intensity laser. No activity was detected in glomeruli or cells (red traces in **B**$_2$). (**B**$_1$; right) Stimulation with a 48 mW laser caused visible, strong calcium signals in multiple glomeruli and surrounding cells (orange traces in **B**$_2$). Overall, we detected calcium signals in 0% of WT glomeruli during 16 mW stimulation (52 glomeruli examined; 5 olfactory bulbs), in 16% of WT glomeruli during 32 mW stimulation (25 glomeruli examined; 1 olfactory bulb) and in 20% of WT glomeruli during 48 mW stimulation (84 glomeruli examined; 6 olfactory bulbs). These results indicate that > 16 mW laser stimuli induce non-specific neuronal activity in olfactory sensory neurons, regardless if they express channelrhodopsin or not. The scale bar in **B**$_1$ (right) applies to all three images.
